# Polymorphisms in nitric oxide synthase and endothelin genes among children with obstructive sleep apnea

**DOI:** 10.1186/1755-8794-6-29

**Published:** 2013-09-06

**Authors:** Siriporn Chatsuriyawong, David Gozal, Leila Kheirandish-Gozal, Rakesh Bhattacharjee, Ahamed A Khalyfa, Yang Wang, Wasana Sukhumsirichart, Abdelnaby Khalyfa

**Affiliations:** 1 Department of Pediatrics, Comer Children's Hospital, Pritzker School of Medicine, Biological Sciences Division, The University of Chicago, 900 E, 57th Street, KCBD, 4112, Chicago, IL 60637, USA; 2 Department of Biochemistry, Faculty of Medicine, Srinakharinwirot University, Sukhumvit 23, Bangkok 10110, Thailand

**Keywords:** Children, OSA, Nitric oxide synthase (NOS), Endothelin (EDN), SNP, Polymorphisms

## Abstract

**Background:**

Obstructive sleep apnea (OSA) is associated with adverse and interdependent cognitive and cardiovascular consequences. Increasing evidence suggests that nitric oxide synthase (*NOS*) and endothelin family (*EDN*) genes underlie mechanistic aspects of OSA-associated morbidities. We aimed to identify single nucleotide polymorphisms (SNPs) in the *NOS* family (3 isoforms), and *EDN* family (3 isoforms) to identify potential associations of these SNPs in children with OSA.

**Methods:**

A pediatric community cohort (ages 5–10 years) enriched for snoring underwent overnight polysomnographic (NPSG) and a fasting morning blood draw. The diagnostic criteria for OSA were an obstructive apnea-hypopnea Index (AHI) >2/h total sleep time (TST), snoring during the night, and a nadir oxyhemoglobin saturation <92%. Control children were defined as non-snoring children with AHI <2/h TST (NOSA). Endothelial function was assessed using a modified post-occlusive hyperemic test. The time to peak reperfusion (Tmax) was considered as the indicator for normal endothelial function (NEF; Tmax<45 sec), or ED (Tmax≥45 sec). Genomic DNA from peripheral blood was extracted and allelic frequencies were assessed for, *NOS1* (209 SNPs), *NOS2* (122 SNPs), *NOS3* (50 SNPs), *EDN1* (43 SNPs), *EDN2* (48 SNPs), *EDN3* (14 SNPs), endothelin receptor A, *EDNRA*, (27 SNPs), and endothelin receptor B, *EDNRB* (23 SNPs) using a custom SNPs array. The relative frequencies of NOS-1,-2, and −3, and *EDN-1*,-*2,-3*,-*EDNRA*, and-*EDNRB* genotypes were evaluated in 608 subjects [128 with OSA, and 480 without OSA (NOSA)]. Furthermore, subjects with OSA were divided into 2 subgroups: OSA with normal endothelial function (OSA-NEF), and OSA with endothelial dysfunction (OSA-ED). Linkage disequilibrium was analyzed using Haploview version 4.2 software.

**Results:**

For NOSA vs. OSA groups, 15 differentially distributed SNPs for *NOS1* gene, and 1 SNP for NOS3 emerged, while 4 SNPs for *EDN1* and 1 SNP for both *EDN2* and *EDN3* were identified. However, in the smaller sub-group for whom endothelial function was available, none of the significant SNPs was retained due to lack of statistical power.

**Conclusions:**

Differences in the distribution of polymorphisms among *NOS* and *EDN* gene families suggest that these SNPs could play a contributory role in the pathophysiology and risk of OSA-induced cardiovascular morbidity. Thus, analysis of genotype-phenotype interactions in children with OSA may assist in the formulation of categorical risk estimates.

## Background

Obstructive sleep apnea (OSA), is the most prevalent form of sleep disordered breathing both in adults and children [1-3] and has been associated with significant neurocognitive, metabolic, and cardiovascular morbidities [4-8]. OSA is characterized by episodes of total and/or partial collapse of the upper airway alternating with normal breathing during sleep, leading to chronic intermittent hypoxia and hypercapnia, sleep fragmentation and increased swings in intrathoracic pressures. This condition may affect 1– 3% of healthy school-aged children [2]. There is accumulating evidence that OSA is strongly linked to cardiovascular morbidity independent of obesity [9-11]. The presence of altered endothelial function is currently viewed as an early risk marker of cardiovascular disease, and is a relatively common occurrence in both adult and pediatric patients with OSA [12-14], and can precede the onset of hypertension [15]. However, not every child with OSA will develop ED, suggesting that genetic factors may play a role.

Nitric oxide synthase (*NOS*) is encoded by three distinct genes, namely neuronal NOS (*nNOS, NOS1*), inducible NOS (*iNOS, NOS2*), and endothelial NOS (*eNOS, NOS3*), which are located on chromosomes 12, 17 and 7, respectively. Studies have examined the possibility that SNPs in these genes may influence their expression and functional activity, and potentially alter the predisposition to cardiovascular disease [16-18]. Accordingly, single nucleotide polymorphisms (SNPs) have been identified in NOS genes, and their association with coronary artery disease, hypertension, and diabetes has been explored [19-22]. Considering the potential role of these enzymes in either OSA or its downstream adverse consequences, it is somewhat surprising that the potential associations between NOS polymorphisms and OSA remain thus far unexplored.

The endothelins (*EDN*) are a family of endothelium-derived peptides that possess vasoconstrictor properties, and are important mediators of both physiological and pathophysiologic processes [23]. The genes encoding for *EDN-1,-2* and*-3* are located on chromosomes 6, 1, and 20, respectively [24]. Several studies have been identified various SNPs on *EDN* genes and also in the genes encoding for their cognate receptors (*EDNRA* and *EDNRB*), and some of these gene variants have been associated with altered susceptibility and prognosis of diseases such as heart failure, dilated cardiomyopathy, diabetic retinopathy, and atherosclerosis [25-31]. Furthermore, genetic polymorphisms in the endothelin-receptor-subtype-A (*EDNRA*) gene have been identified as conferring increased susceptibility for OSA in adults [32].

Based on aforementioned considerations, we hypothesized that single nucleotide polymorphisms (SNPs) in *NOS-* and *EDN*-related genes in children may contribute to the risk of pediatric OSA or its downstream vascular consequences.

## Methods

### Subjects

The study was approved by the University of Louisville Human Research Committee, and informed consent was obtained from the legal caretaker of each participant. Consecutive healthy pre-pubertal children (ages 5–10 years) were recruited from the community, and the cohort was enriched for the presence of habitual snoring. All children underwent a standard polysomnographic evaluation in the sleep laboratory at the University of Louisville Pediatric Sleep Laboratory, after which assessment of endothelial function (when possible) and a blood draw were performed between 7:00 to 8:00AM in fasting conditions.

### Overnight polysomnography

A standard overnight multichannel polysomnographic evaluation was performed in the sleep laboratory as described previously [33]. Sleep architecture was assessed by standard techniques [34]. The proportion of time spent in each sleep stage was expressed as percentage of total sleep time (%TST). Obstructive apnea was defined as the absence of airflow with continued chest wall and abdominal movement for duration of at least two breaths [33]. Hypopneas were defined as a decrease in oronasal flow of ≥50% with a corresponding decrease in SpO_2_ of ≥4% and/or arousal [33]. The obstructive apnea/hypopnea index was defined as the number of apneas and hypopneas per hour of TST. Arousals were defined as recommended [35] and included respiratory-related (occurring immediately following an apnea, hypopnea, or snore), technician-induced, and spontaneous arousals. Arousals were expressed as the total number of arousals per hour of sleep time (arousal index). Control children required the presence of an AHI<2 in the absence of a history of snoring as well as no snoring detected during the sleep study. Habitually snoring children with AHI>2/hrTST and a nadir oxyhemoglobin saturation <92% were considered to have OSA [33].

### Body mass index

Children were weighed using the InBody 320 scale (Biospace; Cerritos, CA), and height (to 0.1 cm) was measured using a stadiometer (Holtain, Crosswell, UK). The BMI was calculated and the BMI *z*-score was computed using US Centers for Disease Control and Prevention 2000 growth standards (http://www.cdc.gov/growthcharts/) and online software (http://wwwn.cdc.gov/epiinfo/). A BMI *z*-score > 1.65 (> 95th percentile) was considered as fulfilling obesity criteria.

### Sphygmomanometry

All children had arterial blood pressure measured noninvasively using an automated mercury sphygmomanometer (Welch Allyn; Skaneateles Falls, New York) at the brachial artery, using the appropriate cuff size on the non-dominant arm.[36] Systolic BP and diastolic BP indices were calculated by dividing the average systolic and diastolic pressure by the respective 95th percentile for BP using National Heart, Lung and Blood Institute guidelines http://www.nhlbi.nih.gov/guidelines/hypertension/child_tbl.htm), computed for age, sex, and height. Hypertension was defined when the SBPi or DBPi was > 1.

### Endothelial function tests

Endothelial function was assessed upon awakening from the sleep study in the morning, using a modified hyperemic test after cuff-induced occlusion of the radial and ulnar arteries as previously described [14,15,37,38]. Briefly, a laser Doppler sensor (Periflux 5000 System, Perimed, Jarfalla, Sweden) was applied over the volar aspect of the hand at the 1st finger distal metacarpal surface and the hand was gently immobilized. Once cutaneous blood flow over the area became stable, the pressure within an inflatable cuff placed at the forearm and connected to a computer-controlled manometer was raised to 200 mmHg for 60 sec during which blood flow was reduced to undetectable levels. The cuff was rapidly deflated and the laser Doppler measured hyperemic responses. As previously shown, the time to peak regional blood flow after occlusion release (Tmax) is highly reproducible and is representative of the post-occlusion hyperemic response, an index of endothelial function [39]. A Tmax value ≥45 sec was considered as the criterion for abnormal endothelial function as previously described [9,10,14,15,37,38].

### DNA extraction

Peripheral blood samples were collected in vacutainer tubes containing EDTA (Becton Dickinson, Franklin Lakes, NJ, USA). All DNA samples were extracted using QIAmp DNA blood kit (Qiagen, Valencia, CA) according the manufacturer’s protocol. The concentration and quality of the DNA were determined using a ND-1000 Spectrophotometer (Nanodrop Technologies, Wilmington, DE, USA). The precise length of genomic DNA was determined by gel electrophoresis using 1% agarose gel. All the purified samples were stored at −80°C until further analyses.

### Custom cardiovascular gene SNP array

The IBC array was developed using SNP and linkage disequilibrium information from the HapMap as well as data from Seattle SNPs, and National Institute of Environmental Health Sciences (NIEHS) SNPs [40]. Briefly, the IBC array contains about 50,000 SNPs from genetic diversity across approximately 2100 genes related to cardiovascular, inflammatory, hemostasis/coagulation, and metabolic phenotypes and pathways. Among those genes, we selected the NOS genes which include *NOS1* (209 SNPs), *NOS2* (122 SNPs) and *NOS3* (50 SNPs). Furthermore, we selected the EDN and EDN receptor genes family which includes EDN1 (43 SNPs), *EDN2* (48 SNPs), *EDN3* (14SNPs), *EDNRA* (27 SNPs) and *EDNRB* (23 SNPs). SNPs were clustered into genotypes with the Illumina Beadstudio software and subjected to quality-control filters at the sample and SNP levels separately within each cohort. Samples were excluded for individual call rates <90%, gender mismatch, and duplicate discordance. SNPs were removed for call rates <95% or Hardy-Weinberg Equilibrium p <10^−7^ in controls from each cohort (regardless of ethnicity). Due to the low-frequency SNPs included in the design and the aim to capture low-frequency variants of large effect across the large dataset, we filtered only on minor allele frequency (MAF) < 0.005.

### Total RNA isolation

Fasting peripheral blood samples were drawn from children within the first hour after awakening and collected in PAXgene Blood RNA tubes (Becton Dickinson, UK). Total RNA was isolated using PAXgene Blood RNA Kit and treated with DNase I (QIAGEN, CA), according to the manufacturer’s protocol. The RNA quantity and integrity were determined using a Nanodrop Spectrophotometer and Agilent 2100 Bioanalyzer Nano 6000 LabChip assay (Agilent Technologies, Santa Clara, CA).

### qPCR validation

Quantitative real-time PCR (QRT-PCR) were performed using the ABI 7500 instrument (Applied Biosystems, Foster City, CA). Complementary DNA was synthesized using a High-Capacity cDNA Archive Kit (Applied Biosystems, Foster City, CA). Five hundred nanograms (500 ng) of total RNA from NOSA and OSA samples were used to generate cDNA templates for RT-PCR with primer specific for EDN1 gene. The TaqMan® Master Mix Reagent Kit (Applied Biosystems, Foster City, CA) was in 25 μl reactions. Various negative controls were included in the PCR reaction to ensure specific amplification. Triplicate PCR reactions were performed in 96-well plates for each gene in parallel with the 18S rRNA. The steps involved in the reaction program included: the initial step of 2 minutes at 50°C; denaturation at 95°C for 10 min, followed by 45 thermal cycles of denaturation (15 seconds at 95°C) and elongation (1 min at 60°C). The expression values were obtained from the cycle number (*Ct* value) using the Biosystems analysis software. The threshold cycle (*C*_T_) values were averaged from each reaction, and each gene was normalized to the 18S rRNA level. These Ct values were averaged and the difference between the 18S Ct (Avg) and the gene of interest Ct (Avg) was calculated (Ct-diff). The relative expression of the gene of interest was analyzed using the 2^-ΔΔCT^ method [41]. Quantitative results are expressed as the mean ±standard deviation (SD). Statistical significance was evaluated by the Student’s *t*-test.

### Statistical analysis

All analyses were conducted using SPSS software (version 19.0; SPPS Inc., Chicago, Ill.), and data are presented as mean ± SD. The association analysis was assessed by using Pearson’s chi-square test implemented in SPSS. A P-value < 0.05 was considered statistically significant for all analyses. Odd ratio and 95% confidence interval were calculated for the minor allele of each SNP. The Haploview version 4.2 software (http://hppt://www.borad.mit.edu/mpg/haploview) was used to analyze the linkage disequilibrium structure, calculating D’ to define haplotype block [42] and to estimate haplotype frequencies. Additionally, pair-wise linkage disequilibrium (LD) among the SNPs was examined using Lewontin’s standardized coefficient D’ and LD coefficient r2 [43], and haplotype blocks were defined according to the method of Gabriel et al. [42] in Haploview 4.2 with default settings. Haplotypes within these blocks were estimated using the estimation of maximization algorithm [44]. The associations derived from the comparisons across OSA and NOSA were assessed in terms of odds ratios (OR) both unadjusted and adjusted for age, gender, ethnicity, and obesity with the corresponding 95% confidence interval (CI).

## Results

**Figure 1:**
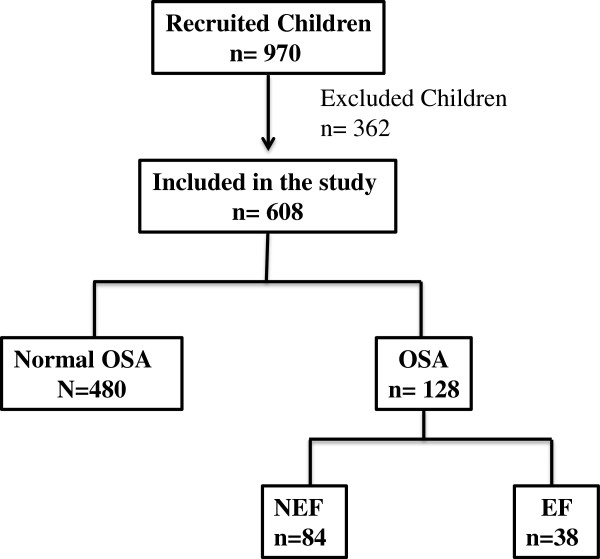
**Schema illustrating the recruitment process in this study.** Children were matched for age, gender and ethnicity. Children were excluded from the study, if they had any chronic medical conditions such as known genetic syndromes, severe asthma or allergies, or if they were on any chronic medications.

The recruitment process of children in this study is shown in Figure 1. As indicated in Figure 1, 970 subjects were recruited, and 362 were excluded from the study because they had chronic medical conditions, such as known genetic syndromes, severe asthma or allergies, or they were receiving chronic medications. A total of 608 children were therefore included, and divided into two groups based on their apnea-hypopnea index (AHI) results in the sleep study: 480 children were controls (NOSA) and 128 children fulfilled the criteria for OSA (OSA) based on AHI. The demographic characteristics and polysomnographic findings of children with OSA and NOSA groups are shown in Table 1, and show markedly similar age, gender, and ethnic distribution indicating that the 2 groups were overall matched for these characteristics. As would be expected based on category attribution criteria, the AHI, apnea index and arousal index were all significantly higher in the OSA group (P<0.0001). Furthermore, mean SpO2 levels in OSA were significantly lower than NOSA group (P<0.001). Notably, we did not find any significant differences in systemic blood pressure among the 2 groups.

**Table 1:** Demographic characteristics in children with and without OSA

**Variables**	**NOSA**	**OSA**	***P-value***
	**(n=480)**	**(n=128)**	
Age (years)	7.14±1.00	7.04±0.99	0.14
Gender (% male)	58.9	55.5	
Ethnicity			
White Caucasian %	72	63.3	
African American %	28	36.7	
BMI z-score	0.81±1.24	1.23±1.38	0.002
SBP (mmHg)	105.46±10.98	105.86±8.05	0.44
DBP (mmHg)	61.80±7.65	63.96±5.53	0.11
Sleep latency (min)	23.76±23.99	19.45±20.71	0.02
REM latency (min)	151.88±62.77	155.24±80.95	0.33
TST (min)	468.77±44.90	469.28±55.29	0.46
Sleep efficiency (%)	88.79±7.89	89.35±9.80	0.28
Stage 1 (% TST)	6.08±4.61	6.05±5.85	0.48
Stage 2 (% TST)	46.32±12.73	43.62±7.93	0.002
Slow wave sleep (% TST)	27.67±9.43	28.91±8.08	0.07
REM sleep (%TST)	20.68±8.35	20.13±10.87	0.30
AHI (h-1 TST)	0.63±0.49	8.28±9.27	<0.0001
Apnea index (h-1 TST)	0.44±0.96	2.53±4.76	<0.0001
Arousal index (h-1 TST)	10.06±7.25	13.37±7.82	<0.0001
Mean SaO2 (%)	97.21±4.82	96.24±2.23	0.0006
Lowest SaO2 (%)	92.66±3.85	86.02±9.10	<0.0001

**Figure 2:**
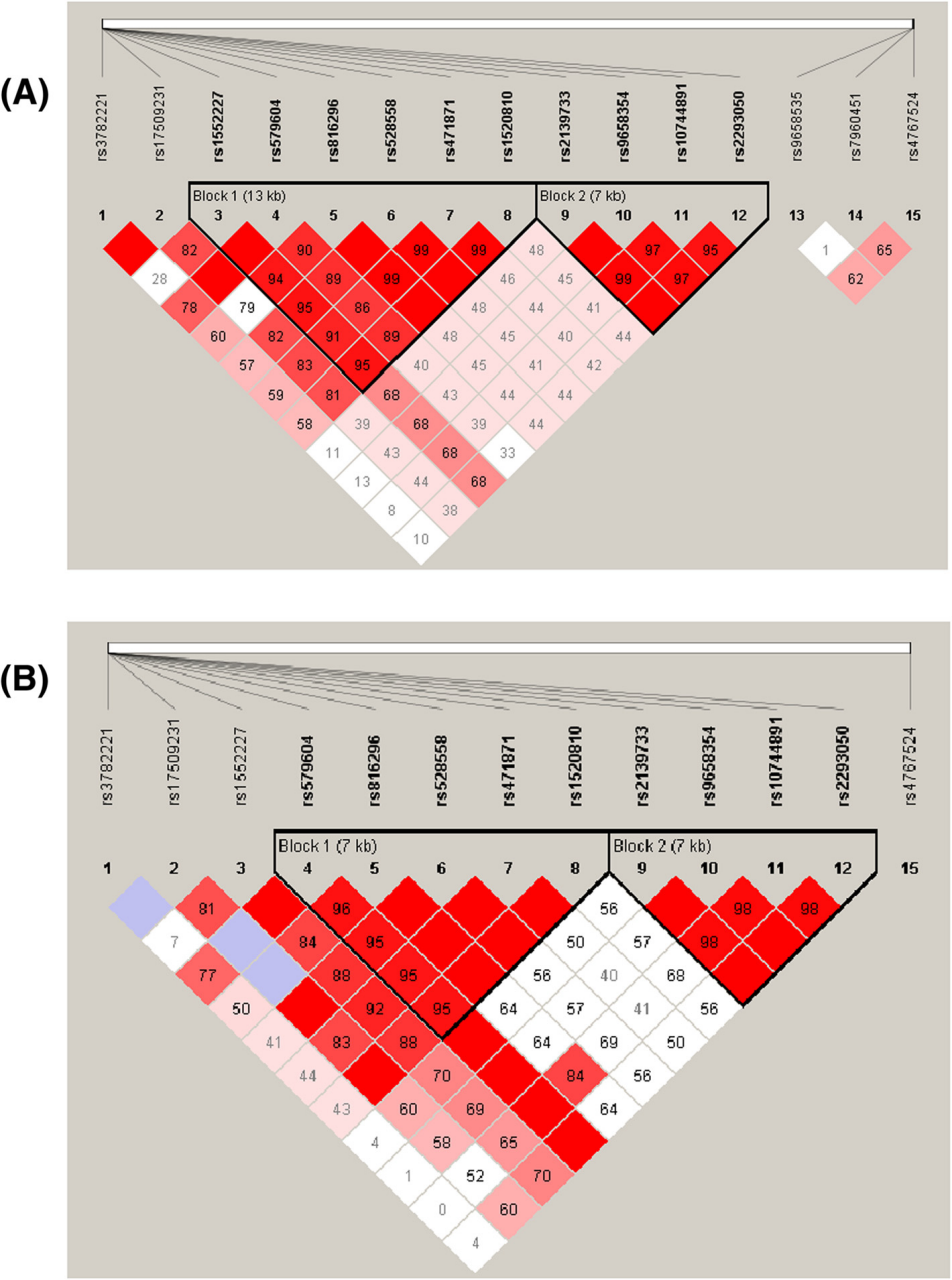
**Pairwise linkage disequilibrium (LD) structure and 15 SNPs of the *NOS1 *gene.** Panel **(A)** represents children without OSA (NOSA), and Panel **(B)** represents children with OSA (OSA). The plot was generated using Haploview 4.2 with D’ Color Scheme (D’=0, D’<1 and D’=1 shown by white, shades of pink and red (respectively) and pairwise r^2^ values shown in diamonds. The value within each diamond represents the pair-wise LD (correlation, measured as D’) between the two SNPs defined by the top left and the top right of the diamond. Solid lines represent SNPs that were used in the haplotype analysis, and are part of the haplotype from SNP block whereas dashed lines represent SNPs that were used in the analysis, but were not part of the haplotype.

**Figure 3:**
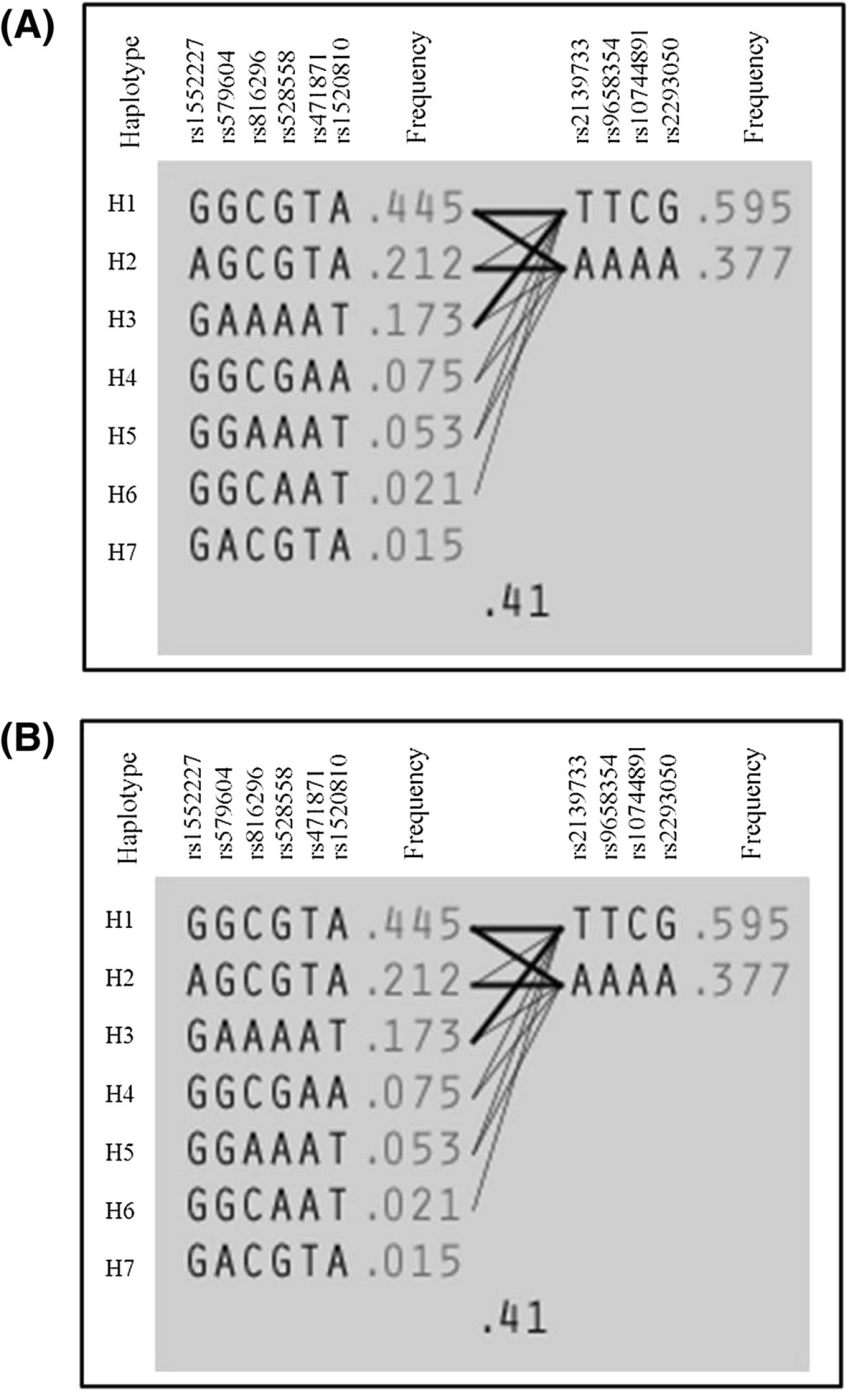
**Haplotype frequencies in children with and without OSA.** Panel **(A)** represents haplotype for children without OSA (NOSA), and Panel **(B)** represents haplotype for children with OSA (OSA).

From a total of 381 SNPs assayed for the 3 *NOS-1*,-*2* and*-3* genes, 15 SNPs in the *NOS1* gene and 1 SNP for *NOS3* gene exhibited statistically significant differences in their frequencies among children with OSA and their matched controls, even after correction for multiple comparisons (Table 2). Linkage disequilibrium (LD) analysis of the 15 SNPs in the *NOS1* gene was assessed for both OSA and NOSA subjects. In NOSA subjects, two haplotype blocks emerged, and are outlined in black triangular regions in Figure 2 (Panel A). In OSA subjects, the haplotype showed the presence of 2 blocks as well (Figure 2, Panel B). The haplotype of these blocks and their frequencies in OSA and NOSA are shown in Figure 3, Panels A and B, respectively. Taken together, the patterns of LD and haplotype frequencies differed between OSA and NOSA, suggesting that some of these SNPs may contribute to OSA risk.

**Table 2:** Distributions of allele and genotype frequencies of NOS SNPs in children with and without OSA

**Gene**	**SNP**	**Allele**	**NOSA**		**OSA**		**P-value**	**OR**	**CI 95%**
NOS1	rs9658535	A/G	n=477		n=128		0.04	0.26	0.07–1.06
			n	%	n	%			
		AA	421	88	116	91			
		GA	52	11	8	6			
		GG	4	1	4	3			
		Allele A	894	94	240	94			
		Allele G	60	6	16	6			
	rs7960451	C/T	n=480		n=128		0.02	0.21	0.05–0.78
			n	%	n	%			
		CC	417	87	112	87			
		TC	59	12	11	9			
		TT	4	1	5	4			
		Allele C	893	93	235	92			
		Allele T	67	7	21	8			
	rs4767524	C/G	n=478		n=128		0.04	0.71	0.43–1.15
			n	%	n	%			
		CC	160	33	52	41			
		GC	242	51	49	38			
		GG	76	16	27	21			
		Allele C	562	59	153	60			
		Allele G	394	41	103	40			
	rs2293050	A/G	n=478		n=128		0.0002	1.59	1.04–2.43
			n	%	n	%			
		AA	82	17	13	10			
		AG	208	44	78	61			
		GG	188	39	37	29			
		Allele A	372	39	104	41			
		Allele G	584	61	152	59			
	rs10744891	G/T	n=465		n=121		0.004	1.72	0.90–3.28
			n	%	n	%			
		GG	185	40	35	29			
		TG	206	44	74	61			
		TT	74	16	12	10			
		Allele G	576	62	144	60			
		Allele T	354	38	98	40			
	rs9658354	A/T	n=478		n=126		0.001	1.74	1.13–2.68
			n	%	n	%			
		AA	80	17	13	10			
		AT	211	44	79	63			
		TT	187	39	34	27			
		Allele A	371	39	105	42			
		Allele T	585	61	147	58			
	rs2139733	A/T	n=478		n=128		0.002	1.65	1.08–2.52
			n	%	n	%			
		AA	77	16	13	10			
		AT	209	44	78	61			
		TT	192	40	37	29			
		Allele A	363	38	104	41			
		Allele T	593	62	152	59			
	rs1520810	A/T	n=478		n=127		0.03	0.86	0.38–1.94
			n	%	n	%			
		AA	263	55	85	67			
		TA	189	40	34	27			
		TT	26	5	8	6			
		Allele A	715	75	204	80			
		Allele T	241	25	50	20			
	rs471871	A/T	n=478		n=127		0.01	0.55	0.37–0.82
			n	%	n	%			
		AA	51	11	13	10			
		AT	212	44	38	30			
		TT	215	45	76	60			
		Allele A	314	33	64	25			
		Allele T	642	67	190	75			
	rs528558	A/G	n=479		n=128		0.03	0.61	0.41–0.92
			n	%	n	%			
		AA	26	5	8	6			
		AG	191	40	35	27			
		GG	262	55	85	66			
		Allele A	243	25	51	20			
		Allele G	715	75	205	80			
	rs816296	A/C	n=478		n=127		0.04	0.58	0.38–0.89
			n	%	n	%			
		AA	24	5	4	3			
		AC	174	36	33	26			
		CC	280	59	90	71			
		Allele A	222	23	41	16			
		Allele C	734	77	213	84			
	rs579604	C/T	n=479		n=128		0.04	N/A	N/A
			n	%	n	%			
		CC	310	65	96	75			
		TC	158	33	32	25			
		TT	11	2	0	0			
		Allele C	778	81	224	88			
		Allele T	180	19	32	12			
	rs1552227	C/T	n=479		n=128		0.02	0.47	0.22–0.97
			n	%	n	%			
		CC	294	61	63	49			
		TC	163	34	53	42			
		TT	22	5	12	9			
		Allele C	751	78	179	70			
		Allele T	207	22	77	30			
	rs17509231	C/T	n=478		n=128		0.02	0.40	0.07–2.41
			n	%	n	%			
		CC	395	83	92	72			
		TC	80	17	34	26			
		TT	3	0	2	2			
		Allele C	870	91	218	85			
		Allele T	86	9	38	15			
	rs3782221	A/G	n=477		n=128		0.02	0.61	0.41–0.90
			n	%	n	%			
		AA	36	7	4	3			
		AG	213	45	47	37			
		GG	228	48	77	60			
		Allele A	285	30	55	21			
		Allele G	669	70	201	79			
NOS3	rs1800780	A/G	n=470		n=126		0.05	0.71	0.47–1.09
			n	%	n	%			
		AA	103	22	16	13			
		AG	240	51	67	53			
		GG	127	27	43	34			
		Allele A	446	47	99	39			
		Allele G	494	53	153	61			

From a total of 155 SNPs for the three *EDN-1,-2* and*-3* genes and their associated *EDN* receptors (*EDNRA* and *EDNRB*), there were 4 SNPs in *EDN1*, and 1 SNP in both of *EDN2* and *EDN3*, in which allelic frequencies were significantly altered in children with OSA (Table 3). No differences emerged for *EDN* receptor (*EDNRA*, *EDNRB*) SNPs. The list and the summary of the significant SNPs in both *NOS* and *EDN* genes such as location of these SNPs, percentage of minor allele frequency (%MAF) are shown in Additional file 1: Table S1.

**Table 3:** Distributions of allele and genotype frequencies of EDN SNPs in children with and without OSA

**Gene**	**SNP**	**Allele**	**NOSA**		**OSA**		**P-value**	**OR**	**CI 95%**
EDN1	rs1014505		n=478		n=127		<0.001	1.28	0.85-1.92
		C/G	n	%	n	%			
		CC	62	13	36	29			
		CG	215	45	45	35			
		GG	201	42	46	36			
		Allele C	339	35	117	46			
		Allele G	617	65	137	54			
	rs2070698	C/T	n=480		n=128		0.04	1.73	1.07-2.79
			n	%	n	%			
		CC	102	21	37	29			
		CT	236	49	66	52			
		TT	142	30	25	19			
		Allele C	440	46	140	55			
		Allele T	520	54	116	45			
	rs2248580		n=478		n=128		0.02	1.07	0.72-1.59
		A/C	n	%	n	%			
		AA	60	12	28	22			
		AC	208	44	46	36			
		CC	210	44	54	42			
		Allele A	328	34	102	40			
		Allele C	628	66	154	60			
	rs2070699	G/T	n=479		n=128		0.002	0.42	0.25-0.69
			n	%	n	%			
		GG	213	45	55	43			
		TG	216	45	45	35			
		TT	50	10	28	22			
		Allele G	642	67	155	61			
		Allele T	316	33	101	39			
EDN2	rs11210273	C/T	n=478		n=128		0.02	NA	NA
			n	%	n	%			
		CC	411	86	105	82			
		TC	67	14	21	16			
		TT	0	0	2	2			
		Allele C	889	93	231	90			
		Allele T	67	7	25	10			
EDN3	rs6064764	C/T	n=479		n=127		0.02	0.90	0.60-1.34
			n	%	n	%			
		CC	27	6	15	12			
		CT	174	36	35	27			
		TT	278	58	77	61			
		Allele C	228	24	65	26			
		Allele T	730	76	189	74			

**Figure 4:**
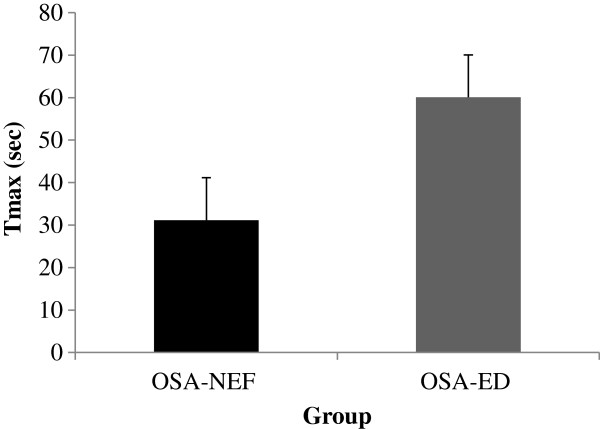
Individual Tmax values in children with OSA and normal endothelial function (OSA-NEF), and children with OSA with endothelial dysfunction (OSA-ED).

Next, we divided OSA subjects into 2 subgroups based on their individual Tmax values, when such values were available: OSA with normal endothelial function (OSA-NEF), and OSA with endothelial dysfunction (OSA-ED). As shown in Figure 4, Tmax values were significantly higher in the 6 OSA-ED subjects compared to 17 subjects with OSA-NEF (P<0.002).

**Figure 5:**
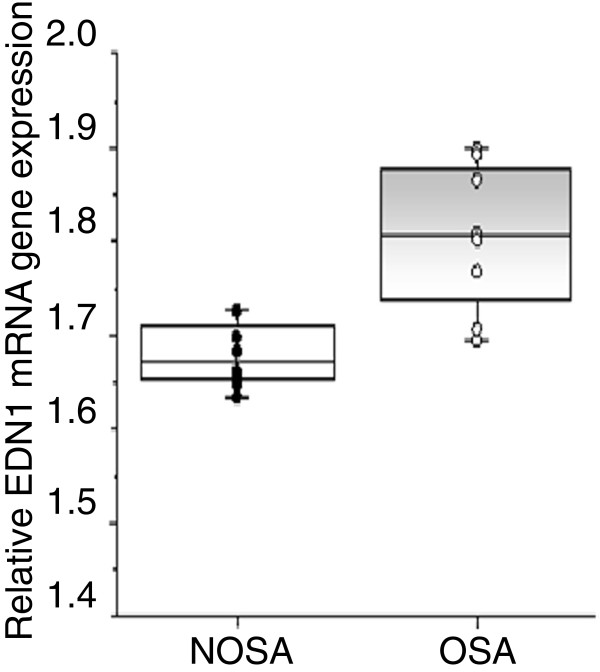
**qRT-PCR analysis for *EDN1 *gene expression in children with OSA and controls (NOSA).** Data are presented as relative mRNA expression levels normalized to 18s, and individual values and boxplots are shown (OSA vs. NOSA: *P*-value <0.0005).

In addition, we quantified the mRNA expression of the *EDN1* gene in 18 matched subjects, 9 with NOSA and 9 with OSA using qRT-PCR. As shown in Figure 5, *EDN1* was significantly increased in children with OSA compared to NOSA (*P*-value 0.0005).

## Discussion

In this study, we report on the allelic frequencies associations of *NOS* and *EDN* gene families in children with and without OSA. The frequency of specific NOS1 and EDN1 SNPs was significantly associated with the presence of OSA, while the frequencies of all other SNPs tested for the *NOS* and *EDN* genes did not show any significant differences between OSA and NOSA. In addition, a subset of the children with OSA showed evidence of endothelial dysfunction even though they were asymptomatic and identified through community-based systematic surveys, thereby confirming previous findings in clinical cohorts on the adverse effect of OSA on endothelial function. Furthermore, in a small subset of children for whom RNA samples were available from peripheral blood monocytes, *EDN1* gene expression was elevated in children with OSA compared to controls.

Before we discuss the potential significance of our findings, some methodological issues deserve comment, in particular subject selection considerations and genetic variances. First, we excluded any child with known diabetes, hypertension, or any other chronic disease condition. This approach could therefore have artificially reduced the magnitude of the association of any given *NOS* of *EDN* allelic variant with OSA. Second, we narrowed the age range of the current cohort such as to minimize as much as possible any confounding factors that might be operational across a wide age range in OSA. Thirdly, closely matched control children are included which should minimize the effect of modifying factors that could be involved in the process of subject selection. In addition, the use of the laser Doppler technique for assessment of vascular responses following cuff-induced arterial occlusion not only permits reproducible determination of the kinetics of post-ischemic reperfusion, but also serves as an accurate reporter of nitric oxide-mediated physiological recruitment of the microvasculature [37,45]. In this context, we also excluded children with a variety of diagnoses that can be associated with endothelial dysfunction [46]. The two important limitations of this study include the relatively small size of the cohort of children studied which could hamper statistical power, and the absence of endothelin plasma level measurements in blood cells for the genes of interest. Although highly desirable, the latter were not possible due to limitations in the amount of blood samples. However, inclusion of the present preliminary findings on endothelial function are intent on further illustrating future directions that will need to encompass specific gene variants to not only the presence or absence of a disorder, i.e., OSA, but also to the presence or absence of a consequence of the disorder, i.e., endothelial dysfunction. We and others have previously shown significant associations between specific gene candidate variances and OSA-associated phenotypes, and this study adds incremental information to potentially significant contributions of *EDN* and NOS gene polymorphisms to this issue [8,47-51]. However, the overall modulatory effects of these polymorphisms to the clinical phenotype of pediatric OSA will have to await more extensive studies involving much larger cohorts. Accordingly, we opted not to implement additional valuable analytical methodologies to derive what we perceive as somewhat premature inferential conclusions from such methods [52,53].

OSA is a multi-factorial and highly prevalent disorder in which both genetic and environmental factors may be involved [54,55]. The role of specific genes that influence the development of OSA is unclear. A precise genetic foundation of OSA has been thus far difficult to identify, because it is still unknown whether some of the putative candidate genes for OSA are directly causal to the expression of the disorder or whether their role in OSA is mediated through other intermediate genes. Similarly, the phenotypic expression of OSA and its consequences is most likely determined by multiple genetic and environmental factors and their interactions. Some of the factors assumed to operate as intrinsic genetic determinants of susceptibility [56-58] have been shown to include inflammatory pathways, lipid membrane transport, and growth factors [58]. Additional external factors that have yet to be corroborated in clinical pediatric cohorts include lifestyle components, such as physical activity, and dietary habits. Previous studies have reported that several single nucleotide polymorphisms might be involved in the pathogenesis of OSA both in adult and children, such as serotonin transporter (*5-HTT*) [59], TNF-α [49,60], fatty acid binding protein 4 [48,61], macrophage migration inhibitory factor [47], NADPH oxidase p22 sub-unit [8,50], and angiotensin I converting enzyme (ACE) [62,63].

In this study, we used 536 SNPs from different genes and pathways to study the association of their allelic frequencies in children with OSA. Here, we show in a total of 209 SNPs assayed with *NOS1* gene, we identified 15 significantly SNPs those allelic frequencies were associated in children with OSA. To the best of our knowledge, no studies were conducted on genetic variance of NOS genes in children with OSA. However, several studies have been reported in human using *NOS* SNPs. For example, several polymorphisms located in *NOS1*, *NOS2*, and *NOS3* genes have been identified; some of these polymorphic sites could be responsible for variations in the genetic control of plasma *NOS* levels, which would be a useful tool for studying the relationship between *NOS* and diseases including asthma [64,65], depressive disorder [66], Parkinson's disease [67], diabetic nephropathy [68], and stroke [69].

The endothelin system consists of G protein–coupled endothelin receptors (*EDNR*) that are activated by endothelin (*EDN*) signaling peptides. Specific interactions between the three different endothelin-subtypes (*EDN-1*, *-2*, *-3*) and the two human endothelin receptors (*EDNRA*, *EDNRB*) are known [70]. Endothelin-1 (*EDN 1*), the most potent vasoconstrictor of the human organism, uses mainly the *EDNRA* as a signal transduction pathway. Our results show different allelic frequencies of *EDN* polymorphisms between OSA subjects and controls. For example, of 155 SNPs for the three *EDN-1*,-*2* and*-3* genes and their associated *EDN* receptors (*EDNRA* and *EDNRB*), there were 4 SNPs in *EDN1*, and 1 SNP in both of *EDN2* and *EDN3*, in which allelic frequencies were significantly altered in children with OSA. We are only aware of a single published report on genetic polymorphisms in endothelin-receptor-subtype-a-gene as a susceptibility factor for adult OSA [32]. These investigators identified 4 candidate SNPs out of 100 in *ENDRA* in patients with OSA, but did not ascertain the significance of their findings by haplotype analysis. Endothelin-1(*EDN1*) is an intercellular signaling molecule expressed in many different organ systems and tissues. Although *EDN1* is best known as a potent vasoconstrictor, *EDN1* also plays important roles in the kidney, nervous system, and in the heart [71]. Furthermore, genetic polymorphisms in the *EDN1* promoter region have been linked to an increased incidence of left ventricular hypertrophy [72], and asthma [73], a known consequence of OSA in children [74]. The increased expression of *EDN1* among children with OSA in the present study would further suggest that genotype-phenotype interactions may indeed be present in pediatric OSA and its cardiovascular morbidities. Indeed, several lines of evidence derived from both clinical studies and animal models have shown that increases in circulating EDN1 in OSA [75-77].

## Conclusion

In conclusion, our results suggest that the *NOS1* and *EDN1* genes may confer an increased risk for the presence of OSA or downstream morbidity. The susceptibility to OSA is a multifactorial process and may result from genetic variants in many genes on different chromosomes. Also, genetic epidemiological studies of biological phenotypes involved in the same pathway can provide relevant information, and can contribute to unravel the mechanisms underlying complex diseases such as OSA. More specifically, the changes in EDN1 gene expression particularly when combined with differences in the distribution of EDN1 polymorphisms, suggest these specific SNPs influence the genetic predisposition to OSA. Thus, analysis of the currently identified EDN1 polymorphism may be useful in the assessment of risk for OSA in a high risk population, such as those children who manifest snoring or have enlarged tonsils and adenoids. Further studies should be carried out to confirm the association reported herein in expanded pediatric cohorts, and tentatively include protein and gene expression levels to enable deciphering the importance and functionality of these genetic factors.

## Competing interests

The authors declare that they have no competing interests.

## Authors’ contributions

SC performed data analysis, and qRT-PCR validation, DG provided the conceptual design of the project, participated in the data analysis and editing final version of the manuscript, L K-G participated in data analysis and sleep studies, RB participated in clinical data, AAK participated in data analysis and haploview, YW reviewed data, WS participated in general discussion and review data, and AK carried data analysis, overall the project and SNPs analysis, writing and editing manuscript. All authors read and approved the final manuscript.

## Pre-publication history

The pre-publication history for this paper can be accessed here:

http://www.biomedcentral.com/1755-8794/6/29/prepub

## Supplementary Material

Additional file 1: Table S1 List of single nucleotide polymorphisms (SNPs) of NOS and EDN genes. Click here for file
